# An improved deep learning method for predicting DNA-binding proteins based on contextual features in amino acid sequences

**DOI:** 10.1371/journal.pone.0225317

**Published:** 2019-11-14

**Authors:** Siquan Hu, Ruixiong Ma, Haiou Wang

**Affiliations:** 1 School of Computer and Communication Engineering, University of Science and Technology Beijing, Beijing, China; 2 Sichuan Jiuzhou Video Technology Co., Ltd, Mianyang, China; 3 School of Chemistry and Biological Engineering, University of Science and Technology Beijing, Beijing, China; Manukau Institute of Technology, NEW ZEALAND

## Abstract

As the number of known proteins has expanded, how to accurately identify DNA binding proteins has become a significant biological challenge. At present, various computational methods have been proposed to recognize DNA-binding proteins from only amino acid sequences, such as SVM, DNABP and CNN-RNN. However, these methods do not consider the context in amino acid sequences, which makes it difficult for them to adequately capture sequence features. In this study, a new method that coordinates a bidirectional long-term memory recurrent neural network and a convolutional neural network, called CNN-BiLSTM, is proposed to identify DNA binding proteins. The CNN-BiLSTM model can explore the potential contextual relationships of amino acid sequences and obtain more features than can traditional models. The experimental results show that the CNN-BiLSTM achieves a validation set prediction accuracy of 96.5%—7.8% higher than that of SVM, 9.6% higher than that of DNABP and 3.7% higher than that of CNN-RNN. After testing on 20,000 independent samples provided by UniProt that were not involved in model training, the accuracy of CNN-BiLSTM reached 94.5%—12% higher than that of SVM, 4.9% higher than that of DNABP and 4% higher than that of CNN-RNN. We visualized and compared the model training process of CNN-BiLSTM with that of CNN-RNN and found that the former is capable of better generalization from the training dataset, showing that CNN-BiLSTM has a wider range of adaptations to protein sequences. On the test set, CNN-BiLSTM has better credibility because its predicted scores are closer to the sample labels than are those of CNN-RNN. Therefore, the proposed CNN-BiLSTM is a more powerful method for identifying DNA-binding proteins.

## Introduction

As a part of the protein family, DNA binding proteins play an important role in RNA editing, methylation and other biological processes. [1]. According to current research, DNA can bind to more than 3% of eukaryotic and prokaryotic proteins [2,3]. In cellular function research, the ability to recognize DNA-binding proteins is a highly meaningful task [4].

In recent decades, some biological experimental approaches have been proposed to discriminate among DNA-binding proteins. For example, "protein blotting" uses SDS-polyacrylamide gel to detect DNA-binding proteins [5]. Hugh et al. identified DNA binding proteins by electrostatic potential and structural units [6]. However, these biological experimental approaches often require considerable time and involve expensive materials; thus, computational methods have advantages compared to experimental methods for identifying DNA binding proteins from massive amounts of data [7].

Computational approaches have advantages in processing sequential data, and there is growing evidence that predicting DNA-binding proteins solely from primary sequences is effective compared with the experimental methods [8–10]. Many computational approaches for predicting DNA binding proteins by primary sequences have been introduced, and machine learning and deep learning methods are among the best.

Models such as the support vector machine (SVM), random forest and other algorithms that belong to the machine learning category have been used to predict DNA binding proteins, [11]. For example, Cai et al. used nonlinear characteristic sets in amino acid sequences to predict DNA-binding proteins using an SVM [12]. Some methods that combine machine learning with mathematical techniques have also been created. For example, by combining the random forest algorithm with the "gray model", Lin et al. created a DNA-binding protein recognizer called iDNA-Prot [13], which reduced the computational time and is thus suitable for large-scale analysis. Wang et al. used normalization, discrete wavelet alteration and cosine alteration to address sequence features, and they used the processed feature set to train an SVM to obtain the predictors of DNA binding proteins [14]. To better capture sequence features, Zou et al. used features from four types of proteins to train an SVM classifier that used three diverse approaches in the eigen transformation process [15]. Rahman et al. proposed a new computational method to identify DNA binding proteins that used a random forest to recognize sequence characteristics and an SVM as the classifier [16]. Chowdhury et al. proposed a new method called iDNAProt-ES, which trains an SVM to obtain a classification model based on the evolutionary messages and structural characteristics of proteins [17]. Liu et al. constructed a modular model framework by combining multi view features and classifiers; the features come from the sequence structure, physical and chemical properties, evolutionary messages and predictive structure messages [18]. This framework can flexibly coordinate different prediction models and perform well on datasets. Adilinaet al. improved the method for extracting sequence features by adopting grouping and recurrent selection to process the obtained feature sets. Their approach reduced the overfitting degree of the model [19]. With the development of network technology, some web implementations for discriminating DNA-binding proteins have been created that can provide online predictive services. One such web site is iDNA-Prot|dis, which incorporated an SVM and linkage information of the primary protein sequence [20] to improve the predictive accuracy of DNA-binding proteins compared to previous methods. Ma et al. designed a more accurate DNA-binding proteins predictor, DNABP, by adopting the random forest algorithm and considering the physical and chemical characteristics of amino acids [21].

The traditional machine learning methods have shown unmatched superiority for solving small-scale data identification problems [22,23]. However, the traditional machine learning methods are difficult to apply to massive samples [24]. Fortunately, the emergence of deep learning has solved the dilemma of traditional machine learning. Deep learning is a new technology based on neural network architecture that has been highly successful at image recognition, voice recognition and many other tasks. [25–27]. Importantly, deep learning can be applied to large amounts of sample data.

Some scholars have perceived the advantages of deep learning methods and applied them to predict DNA-binding proteins [7,28,29]. Delong et al. were the first to show that protein features can be identified by deep learning; this work provided the original idea for the prediction of DNA-binding proteins [28]. Zeng et al. went further, using a convolutional neural network (CNN) to predict DNA-binding proteins [29]. Qinhu et al. proposed a new method based on a CNN combined with instance learning to identify DNA binding proteins; this method fully considers the inherent weak supervision information of sequences to improve the effect [30]. Recently, Qu et al. combined a CNN with a recurrent neural network (CNN-RNN) to predict DNA-binding proteins [7]. Previous work has improved the flexibility with which features of protein sequences can be extracted. Compared with the machine learning methods, the application of deep learning not only made it possible to use millions of protein sequences for model training but also improved the prediction accuracy by approximately 5.9 percentage points.

However, it is worth noting that neither the CNN used in [29] nor the recurrent neural network used in [7] take context into account when processing sequence information. From previous works other than protein sequences, contextual relationship has been found to be important features of sequence information [31]. These considerations inspired us to wonder whether amino acid sequence also contain contextual features. If contextual relationships exist in amino acid sequences, capturing those features might improve the ability to predict DNA-binding proteins.

**Fig 1:**
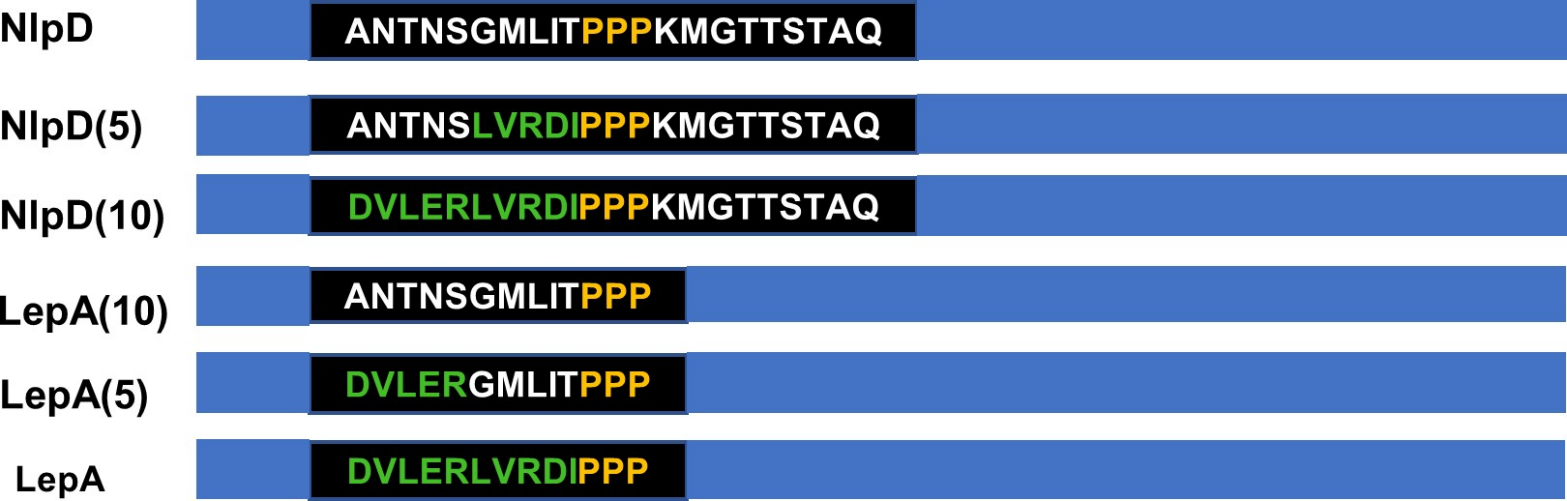
Changes in the context of PPP in both the NlpD and LepA sequences had an important impact on its function.

Regarding the above question, some other studies on the context of amino acid sequences have also inspired us. Ashraf et al. found that the contextual relationships among amino acid sequences are important sequence features and have a positive effect when predicting protein structures [32,33]. The early GOR method has achieved preliminary success in second level architecture prognosis by using alternation statistics based on context and information theory. [34]. Starosta et al. found that a change in the context of consecutive proline triggers (PPP) in both the NlpD and LepA sequences had an important impact on its function, as shown in Fig 1 [35]. These studies imply that contextual relationships do exist in amino acid sequences; thus, exploiting this information will contribute to improving the prediction accuracy of DNA-binding proteins.

Assuming that there is a primary sequence of a protein S = (ALQPGGS…), the contextual features of S can be expressed as follows:

$$F(S)=\sum_{i=1}^{n}\sum_{j=1}^{n}com(S_{i},S_{j})(i\neq j).$$

In the above formula, n represents the length of the sequence, and *S*_*i*_ and *S*_*j*_ represent the i and j elements in the sequence. In addition, *com*(*S*_*i*_,*S*_*j*_) represents the functional affinity scores of the two elements. The affinity scores represent the functional information expressed by amino acids in a specific context. The sum of the affinity scores between different amino acids in the whole sequence constitutes the contextual features.

To capture the contextual features of amino acid sequences, it is helpful to use the bidirectional long short-term memory recurrent neural network (Bi-LSTM), which is a recent deep learning network development [36–38]. A Bi-LSTM effectively captures the contextual information of statements or sequences. which can potentially improve contextual feature extraction and thus achieve a better recognition effect for DNA-binding proteins.

**Fig 2:**
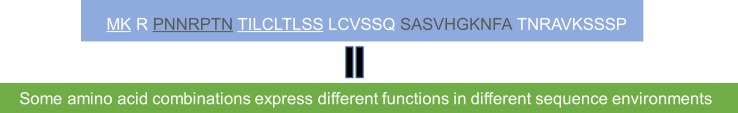
An amino acid sequence can be considered a sentence.

In this paper, we apply a new deep learning model, named CNN-BiLSTM, to identify DNA-binding proteins. The CNN improves model robustness and reduces the error by using the backpropagation algorithm and loss function, while the Bi-LSTM mines the relationships between the contexts in both the forward and backward directions. CNN-BiLSTM includes two convolutional layers, two pooling layers and a Bi-LSTM recurrent neural network layer. In this model, an amino acid can be considered a “word” in a sequence, and multiple amino acids are considered to be a “phrase”. The roles of these “words” or “phrases” are influenced by the context in which they are located, just as a word has different meanings in different contexts, as shown in Fig 2.

Compared with the preceding models for identifying DNA binding proteins, our method captures the contextual features of amino acid sequences and exhibits better performance. The experiments show that our method is more robust than are previous methods in terms of its ability to generalize from the training dataset; consequently, it is more accurate at predicting DNA-binding proteins.

### Materials

The Universal Protein Resource (UniProt) is a repository that contains a large number of protein sequences, and the raw dataset was manually annotated and reviewed [39]. We obtained the sequences of DNA binding proteins from UniProt. Our use of the UniProt website conformed with its terms of use.

In the process of extracting protein data from UniProt, we excluded sequences shorter than 50 and longer than 1,280; we need to limit the input data length to the deep learning model because a longer length requires more computing resources needed. Most of the proteins in the database are in the 50–1280 range. Therefore, to collect data of different lengths effectively, we chose this standard. Finally, we obtained 17,151 positive samples from UniProt, all of which were marked as DNA binding protein sequences. Simultaneously, we obtained 50,000 negative samples from UniProt, none of which were marked as DNA binding protein sequences. For these positive and negative samples, we randomly selected 85% for training and used the remaining 15% as validation sets for model training. (see Table 1 for specific circumstances).

**Table 1:** Unbalanced dataset for model training.

Dataset	Positive	Negative	Total
Original set	17,151	50,000	67,151
Training set (85%)	14,578	42,500	57,078
Validation set (15%)	2,573	7,500	10,073

The number of positive and negative samples in the table above are not equivalent. Therefore, we established a balanced dataset to carry out comparative experiments. The training data include both positive and negative samples of 14,578 protein sequences, and the validation set data included 2,573 protein sequences. The comparative experiment is helpful for investigating the effect of having balanced positive and negative samples on the prediction accuracy. Additionally, to confirm the universality of CNN-BiLSTM, we adopted the datasets used in [40] for testing. In addition, we used 32,000 samples from [7] as training sets to compare the differences in the predictive scores of different models on independent sample sets. (see Table 2 for the specific circumstances).

**Table 2:** Balanced experiment dataset for model training.

Dataset	Positive samples	Negative samples	Total
Original set	17,151	17,151	34,302
Training set	14,578	14,578	29,156
Validationt set	2,573	2,573	5,146
*Arabidopsis*	100	100	200
Dataset from the literature	16,000	16,000	32,000

To test the model generalizability, we have also prepared a tagged test set whose data are not used during model training. This dataset included 500 units with 500 positive and 500 negative samples. At the same time, we also prepared a large test set with 10,000 units to test the model effect on a large dataset. The test results of the model on these sets can reflect the real prediction level of the model. (see Table 3 for the specific circumstances).

**Table 3:** Test set.

Dataset	Positive	Negative	Total
Test set (500)	500	500	1,000
Test set (10,000)	10,000	10,000	20,000

## Methods

Deep learning is both a set of algorithms and a branch of machine learning. Deep learning can approximate complex functions through multiple neural network layers and represent abstract data. Deep learning uses the backpropagation algorithm to update the internal model weights, and deep learning can discover the characteristics of complex data and pass them on to the next layer of the network [41].

A CNN can process data in matrix form well and extract its features effectively through feature mapping [42]. Unlike a CNN, RNN is designed to address sequence information [43]. However, RNNs suffer from time lag problems during training because they often encounter disappearing gradient or gradient explosion problems during training.

### Bidirectional long-term memory recurrent neural network

**Fig 3:**
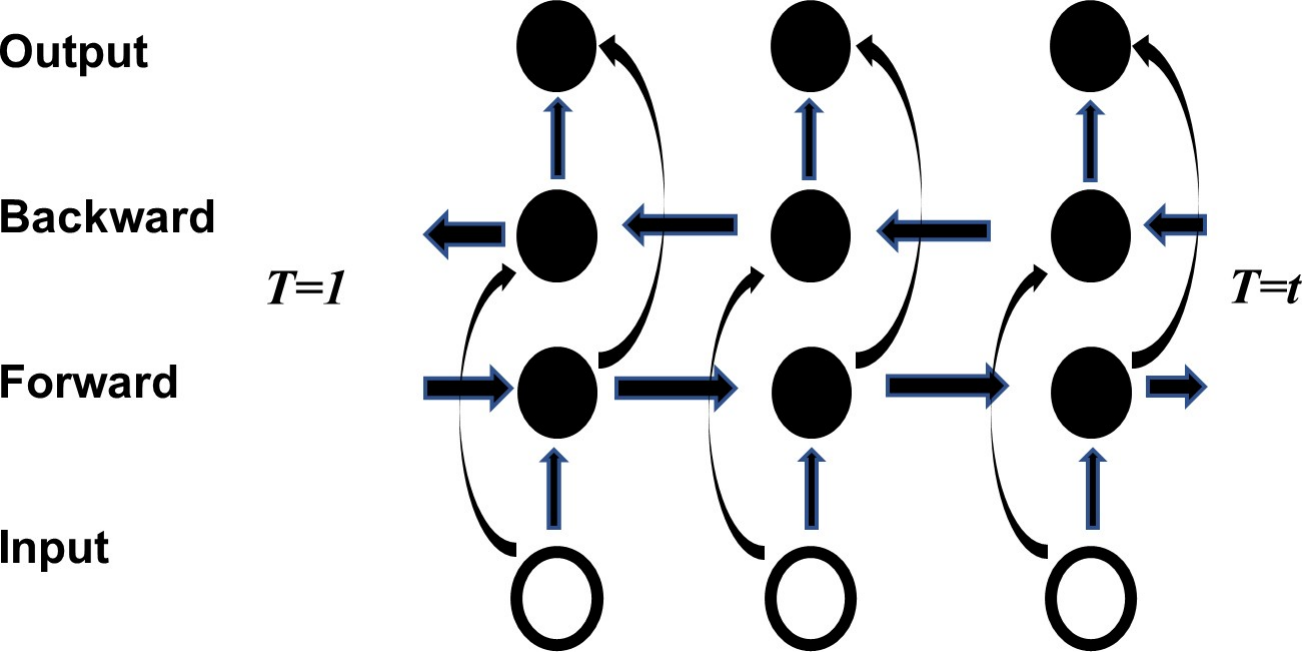
Bi-LSTM structure.

The long short-term memory recurrent neural network, first proposed by Hochreiter & Schmidhuber in 1997, was originally designed to address long time lag problems in RNNs [44]. Sometimes, however, predictions need to be determined by considering both previous and subsequent inputs. Therefore, Zhang et al. proposed the bidirectional long short-term memory network to process sequence information [45]. The network is first calculated forward from time 1 to time t in the forward layer. The output of the hidden layer at each timepoint is obtained and saved, as shown in Fig 3. Then, in the backward layer, the outcome of the hidden layer at each time is obtained and saved by the reverse calculation, from time t to time 1. Finally, the output takes into account the results of both the forward layer and the backward layer.

### Deep learning model

**Fig 4:**
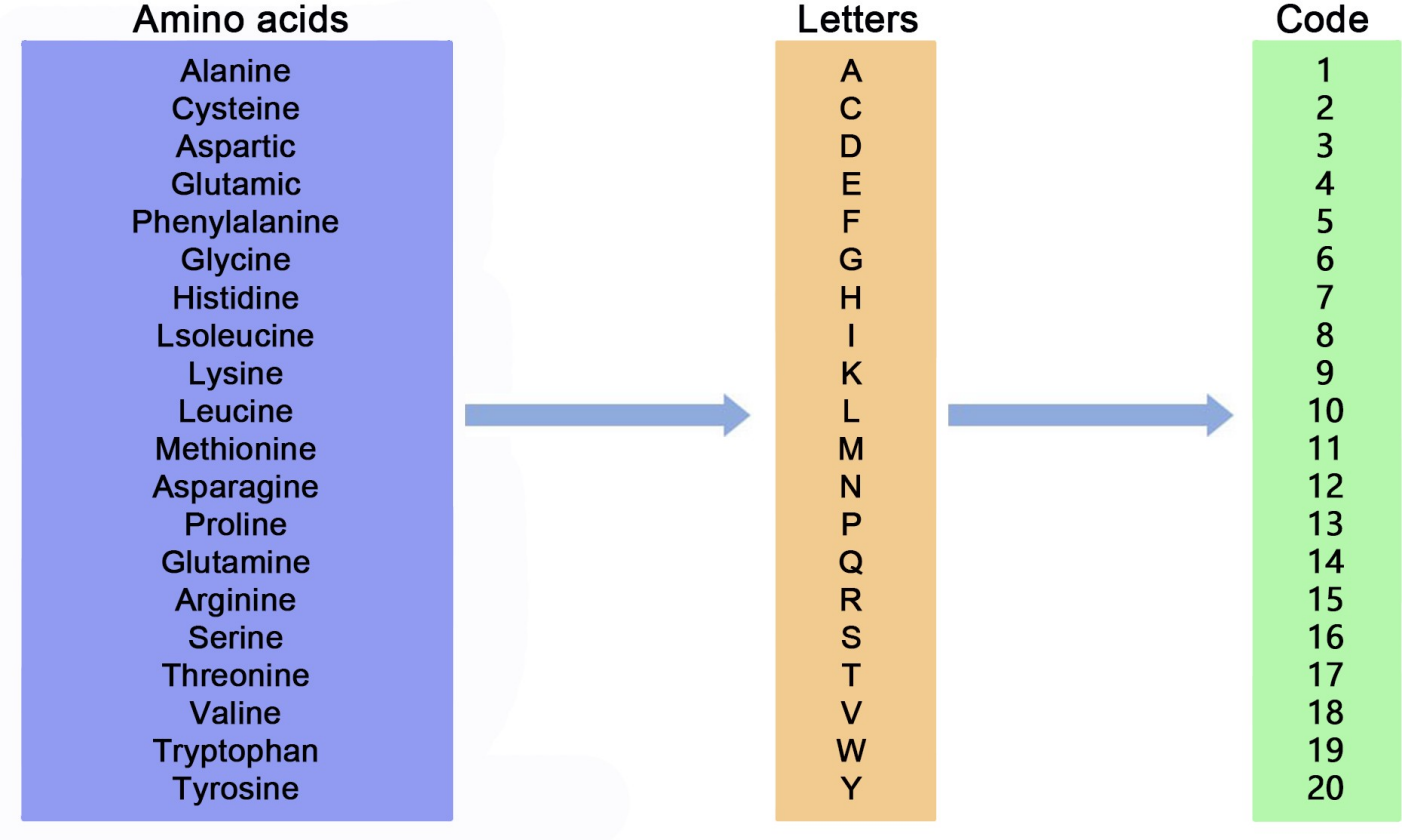
Amino acid encoder.

There are 20 amino acids that make up proteins. Each amino acid is represented by a capital letter [46]. We use different numbers to represent different types of amino acids. (see Fig 4 for details).

**Fig 5:**
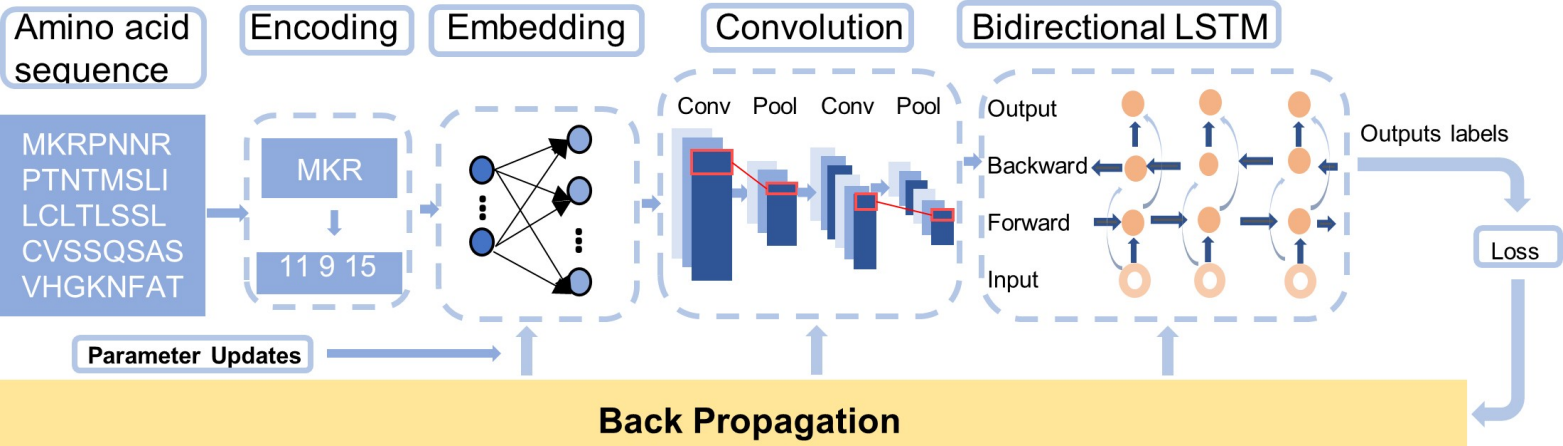
Model.

The deep learning model is composed of the following four parts: a coding layer, an embedding layer, a convolutional layer and a Bi-LSTM layer. The coding layer represents each amino acid as a particular number. The embedding layer translates the amino acid sequences into continuous vectors. The convolution layer consists of two convolutions and two max pooling operations. The goal of the Bi-LSTM is to grasp the contextual features of amino acid sequences. (see Fig 5 for the specific circumstances).

Similar to the use of the deep learning method in the field of image recognition, we use filters in the convolutional layer to obtain the features of protein sequences and further extract their main features in the pooling layer. Then, we utilize the Bi-LSTM to acquire the contextual features of amino acid sequences. In this work, an amino acid sequence is treated as a complete sentence, and each amino acid is treated as a word. We not only capture the characteristic information of the entire sequence but also capture the effects of the contextual features of sequences on amino acid combinations. All the information obtained by our model serves as a predictor of DNA-binding proteins.

### The model execution process

**Fig 6:**
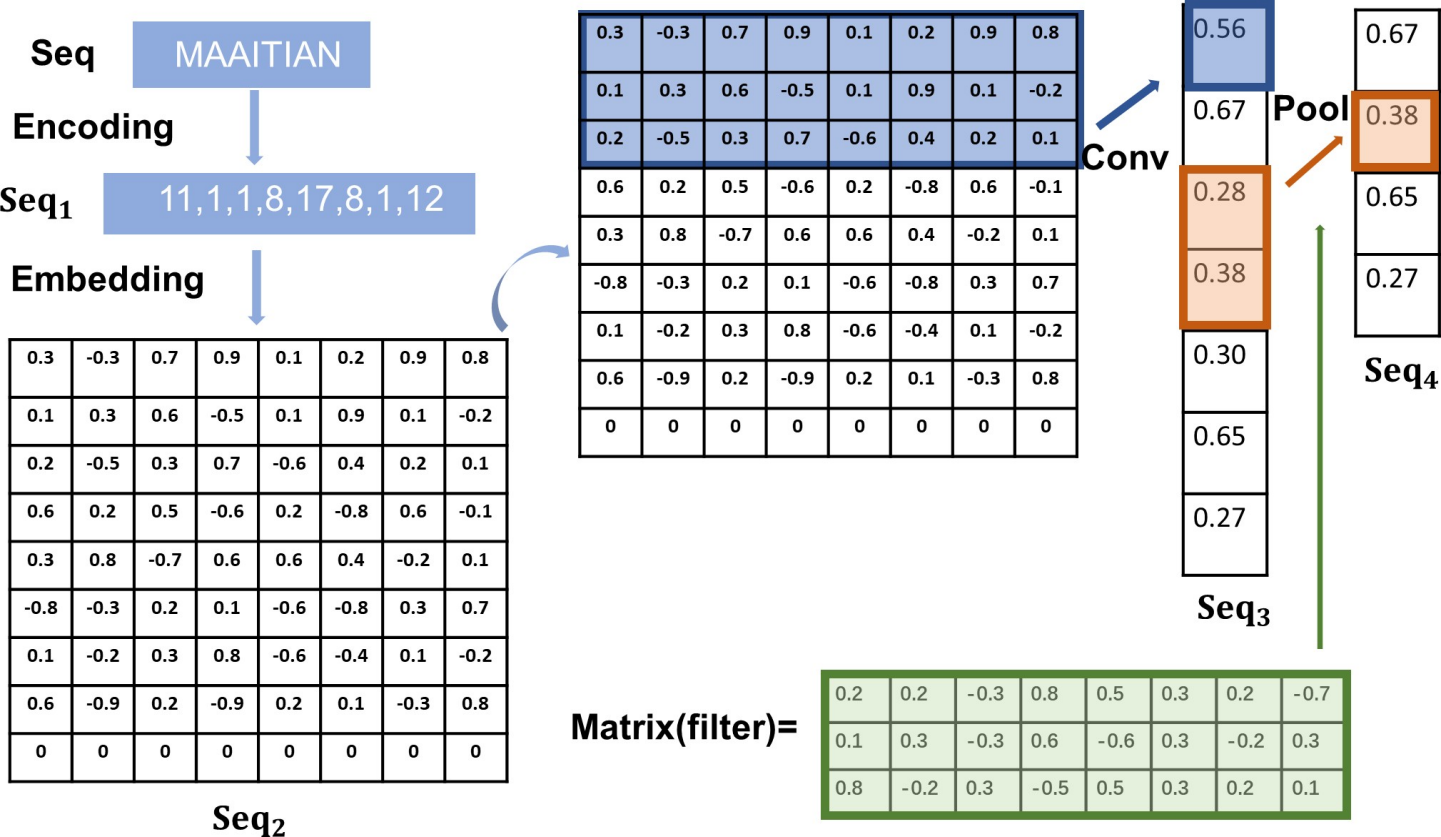
Embedding and convolution.

To clarify the above process, we take the sequence ***Seq = MAAITIAN*** as an example input and illustrate its flow through the layers. (see Fig 6 for details).

Note that the maximum length of protein sequences in the dataset used in this paper is 1,280 because the length range of most of the protein sequences in our dataset is 50–1280. During the coding process, sequences shorter than 1,280 are zero-filled at the end to keep all the coded sequences aligned. To simplify this concept, in Fig 6, we assume that the maximum sequence length is 9.

Thus, during coding, the sequence ***Seq*** becomes a list of numbers after passing through the coding layer, as shown in (1).

$${Seq}_{1}=Encoding(Seq)=(11,1,1,8,17,8,1,12)$$ (1)

Next, the sequence is transformed into a multidimensional matrix, as shown in (2).

$${Seq}_{2}=Embedding({Seq}_{1})\left[\begin{array}{cccccccc} 0.3 & -0.3 & 0.7 & 0.9 & 0.1 & 0.2 & 0.9 & 0.8\\ 0.1 & 0.3 & 0.6 & -0.5 & 0.1 & 0.9 & 0.1 & -0.2\\ 0.2 & -0.5 & 0.3 & 0.7 & -0.6 & 0.4 & 0.2 & 0.1\\ 0.6 & 0.2 & 0.5 & -0.6 & 0.2 & -0.8 & 0.6 & -0.1\\ 0.3 & 0.8 & -0.7 & 0.6 & 0.6 & 0.4 & -0.2 & 0.1\\ -0.8 & -0.3 & 0.2 & 0.1 & -0.6 & -0.8 & 0.3 & 0.7\\ 0.1 & -0.2 & 0.3 & 0.8 & -0.6 & -0.4 & 0.1 & -0.2\\ 0.6 & -0.9 & 0.2 & -0.9 & 0.2 & 0.1 & -0.3 & 0.8\\ 0 & 0 & 0 & 0 & 0 & 0 & 0 & 0 \end{array}\right]$$ (2)

In the convolutional layer, we use the filter matrix to scan ***Seq***_**2**_ and obtain ***Seq***_**3**_, which is also a matrix, as shown in (3) and (4).

$$Matrix(filter)=\left[\begin{array}{cccccccc} 0.2 & 0.2 & -0.3 & 0.8 & 0.5 & 0.3 & 0.2 & -0.2\\ 0.1 & 0.3 & -0.3 & 0.6 & -0.6 & 0.3 & -0.2 & 0.3\\ 0.8 & -0.2 & 0.3 & -0.5 & 0.5 & 0.3 & 0.2 & 0.1 \end{array}\right]$$ (3)

$${Seq}_{3}=Conv({Seq}_{2})=\left[\begin{array}{c} 0.56\\ 0.67\\ 0.28\\ 0.38\\ 0.30\\ 0.65\\ 0.27 \end{array}\right]$$ (4)

In the pooling layer, we adopt the max pooling method. This method adopts the maximum value of two numbers as their representative, as shown in (5).

$${Seq}_{4}=Pool({Seq}_{3})=\left[\begin{array}{c} 0.67\\ 0.38\\ 0.65\\ 0.27 \end{array}\right]$$ (5)

**Fig 7:**
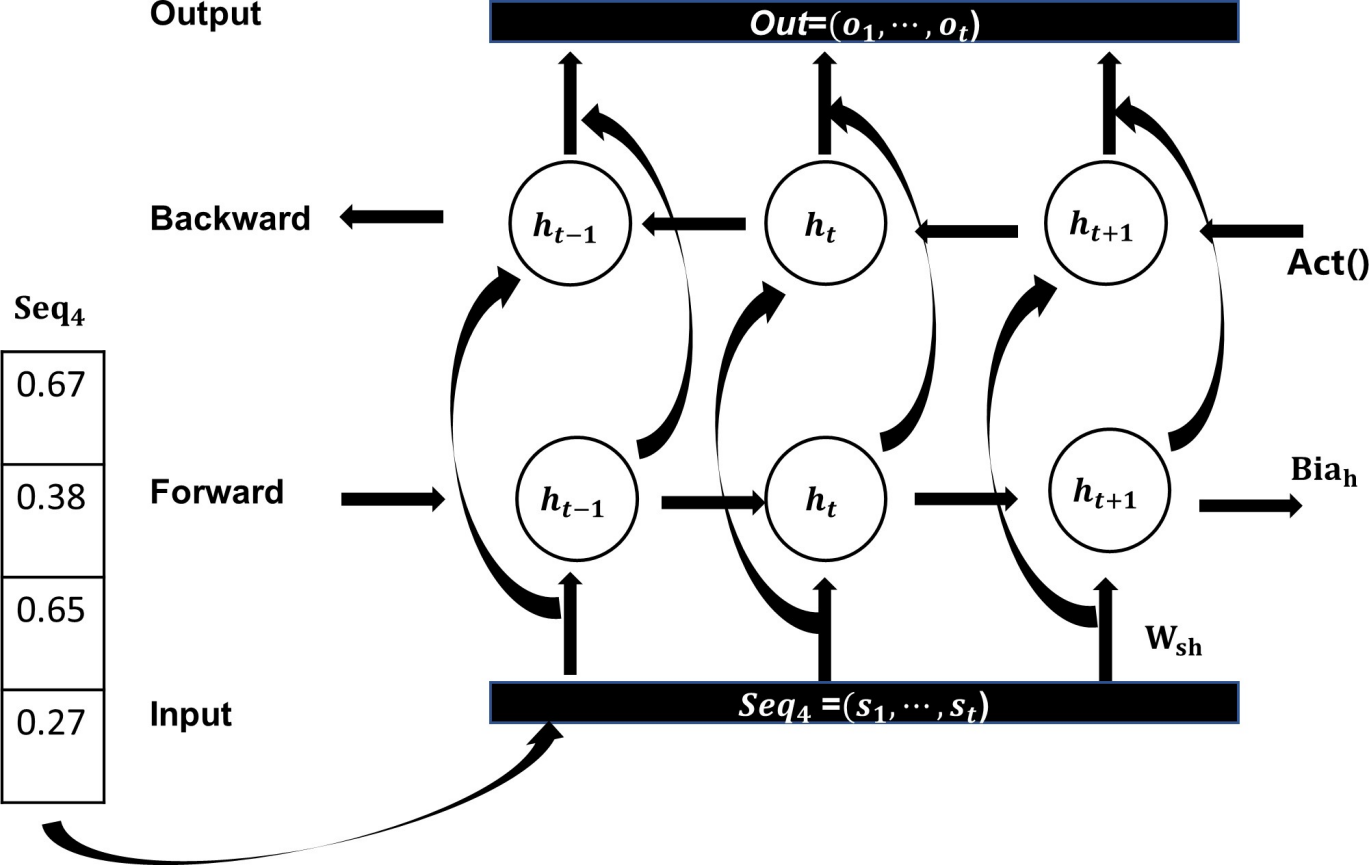
Bi-LSTM layer.

The Bi-LSTM layer computes ***Hid*** = (***h***_**1**_,⋯,***h***_***t***_) and ***Out*** = (***o***_**1**_,⋯,***o***_***t***_) for the input ***Seq***_**4**_ = (***s***_**1**_,⋯,***s***_***t***_), iterating the formulas below from ***t*** = 1 to ***T***, as shown in (6) and (7). (see Fig 7 for details).

$$h_{t}=Act(W_{sh}h_{t}+W_{hh}h_{t-1}+{Bia}_{h})$$ (6)

$$o_{t}=W_{ho}h_{t}+{Bia}_{o}$$ (7)

***W***_***sh***_ is the weight matrix between the input and intermediate layer, ***Bia***_***h***_ is the bias vector for intermediate layer vectors, and ***Act*** is a nonlinear activation function. Finally, for a given sequence ***Seq***, we use the function ***F(Seq)*** to calculate its score to determine whether it is a DNA-binding protein, as shown in (8).

F(Seq)=Bi−LSTM(CNN(Embedding(Encoding(Seq)))) (8)

We implemented the method on the Keras platform [47]. The laboratory protocols for this study are available at (http://dx.doi.org/10.17504/protocols.io.2rdgd26) and that site contains both the tools and the steps required for the experiment. All the source code and data used in this study are available from the Figshare server at (https://doi.org/10.6084/m9.figshare.8131244).

## Results and discussion

### Program architecture

**Fig 8:**
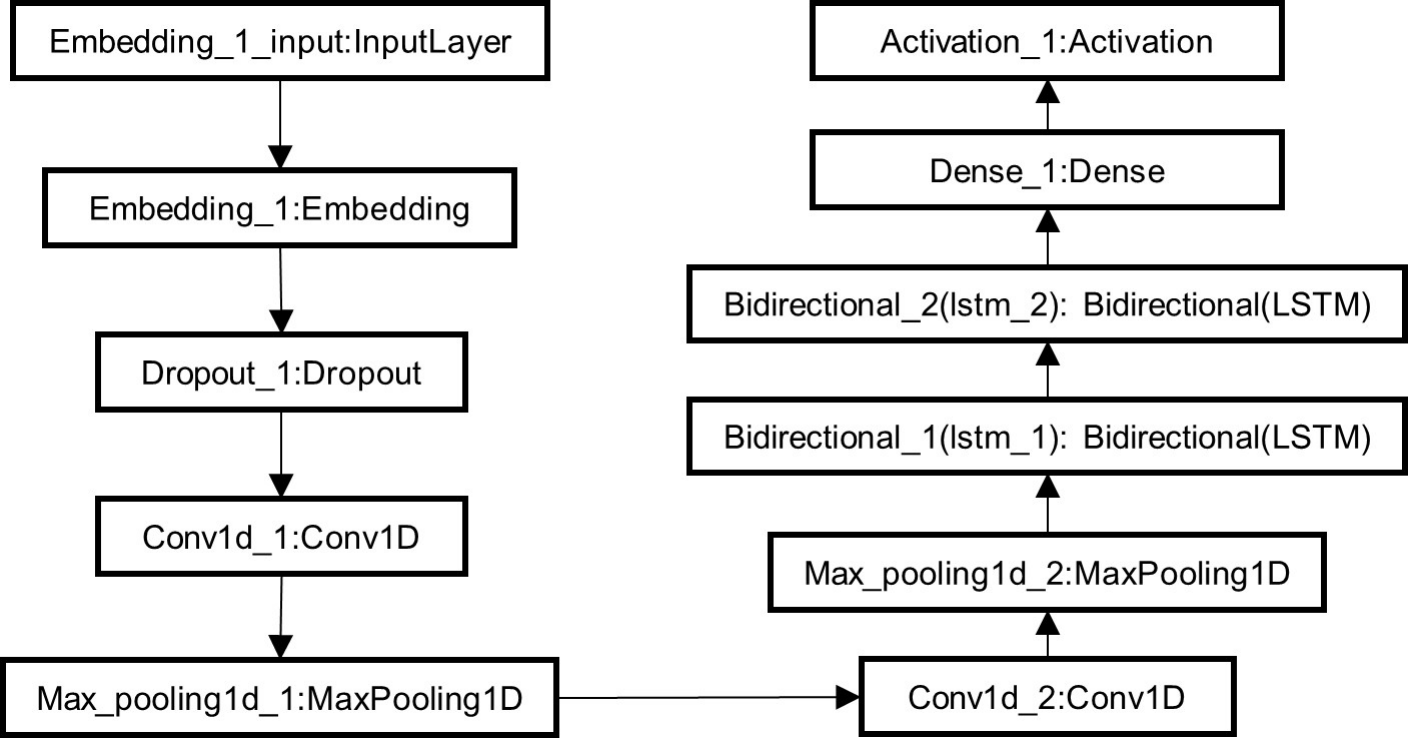
Program architecture.

Based on the method mentioned above, we developed our program on the Keras platform and used its functions to construct our program architecture. (see Fig 8 for details).

### Experimental setup and results

We used both a balanced dataset and unbalanced dataset in our experiments. We also used cross-validation methods to train the model. Here, K represents the proportion of training set data to total data in the training model, while 1-K represents the proportion of the validation set data to the total data. For each experiment, we checked the performance of the model on the validation set under three different conditions: k = 0.85, k = 0.8 and k = 0.9 and found that the model fits the samples better and predicts the sequences more accurately when k = 0.85. (see Table 4 for details).

**Table 4:** Experiment with different training set proportions.

Experimental category	K
Balanced experiment	0.85
	0.8
	0.9
Unbalanced experiment	0.85
	0.8
	0.9

The validation accuracy (Validation-Acc) by the best model of the balanced experiment is 94.6%. The test accuracy (Test-Acc) of the model was 94.1% for the 500-unit test set (500 positive samples and 500 negative samples) and 94.5% for the 10,000-unit test set (10,000 positive samples and 10,000 negative samples). For the unbalanced experimental group, the Validation-Acc of the best model is 96.5%. This model achieved a Test-Acc of 90.4% on the 500-unit test set and 90.7% on the 10,000-unit test set. (see Table 5 for details).

**Table 5:** Validation and test results.

Experimental category	Validation-Acc	Test samples	Test-Acc
Balanced experiment	94.6%	1,000	94.1%
		20,000	94.5%
Unbalanced experiment	96.5%	1,000	90.4%
		20,000	90.7%

In the balanced experiment, the model Test-Acc on the test samples is highly similar to its Validation-Acc. This result shows that the model trained by the balanced dataset exhibits almost no overfitting, and its predictions are both sensitive and accurate. On the unbalanced experiments, the Validation-Acc of the model was higher than that on the balanced experiments, but it performed worse on the test samples. This result indicates that the model trained on the unbalanced data has a slight overfitting phenomenon; thus, its prediction ability is weaker than the model trained on the balanced data.

### Comparison of the results of different models

We also compared other models for predicting DNA binding proteins with the model proposed in this paper [7,21, 48,49]. Judging from the results of different experiments, the Validation-Acc of CNN-BiLSTM is 96. 5%—7.8% higher than that of SVM, 9.6% higher than that of DNABP and 3.7% higher than that of CNN-RNN, respectively. (see Table 6 for details).

**Table 6:** Validation-Acc of different models.

Model category	Validation-Acc
SVM	88.7%
DNABP	86.9%
CNN-RNN	92.8%
CNN-BiLSTM	96.5%

The results in Table 6 show that the proposed CNN-BiLSTM is more capable of capturing protein sequence features and fitting data than are other models.

When tested on the test samples (*Arabidopsis*) in the literature [40], the Test-Acc of CNN-BiLSTM reached 93%—12% higher than that of SVM, 19% higher than that of DNA Binder and 4% higher than that of CNN-RNN. (see Table 7 for details).

**Table 7:** Test-Acc of different models (*Arabidopsis*).

Model categories	Test-Acc
SVM	81.0%
DNABP	89.6%
CNN-RNN	89.0%
CNN-BiLSTM	93.0%

In addition, in order to further verify our model, we used independent test samples in literatures [14,16,17,19] to test our model. Four methods are used to estimate the performance of our method, including Accuracy, Recall, Specificity, and MCC (Mathew’s correlation coefficient). Their expressions are listed below:

$$Accuracy=\frac{TP+TN}{TP+FP+TN+FN}$$ (9)

$$Recall=\frac{TP}{TP+FN}$$ (10)

$$Specificity=\frac{TN}{TN+FP}$$ (11)

$$MCC=\frac{TP*TN-FP*FN}{\sqrt{(TP+FN)*(TN+FP)*(TP+FN)*(TN)*FN}}$$ (12)

Where TP, TN, FP, FN indicates the number of true positive, true negative, false positive and false negative respectively.

In Table 8, our model is compared with the models in the above literatures including: iDNA-Prot [13] Compression technology on PSSM [14], DPP-PseAAC [16], iDNAProt-ES [17].

**Table 8:** The performance of our methods and other existing methods on independent datasets.

Model category	Accuracy	Recall	Specificity	MCC
iDNA-Prot	67.20%	67.7%	66.7%	0.344
Compression technology on PSSM	76.3%	92.5%	60.2%	0.557
DPP-PseAAC	77.4%	83.9%	71.0%	0.553
iDNAProt-ES	80.6%	81.3%	80.0%	0.613
CNN-BiLSTM	81.2%	89.2%	73.1%	0.632

As shown in Table 7 and Table 8, the performance of CNN-BiLSTM on the independent test samples is better than the performances of other models, which indicates that CNN-BiLSTM is more stable and more trustworthy in practical application.

### Comparison of characteristics of different models

**Fig 9:**
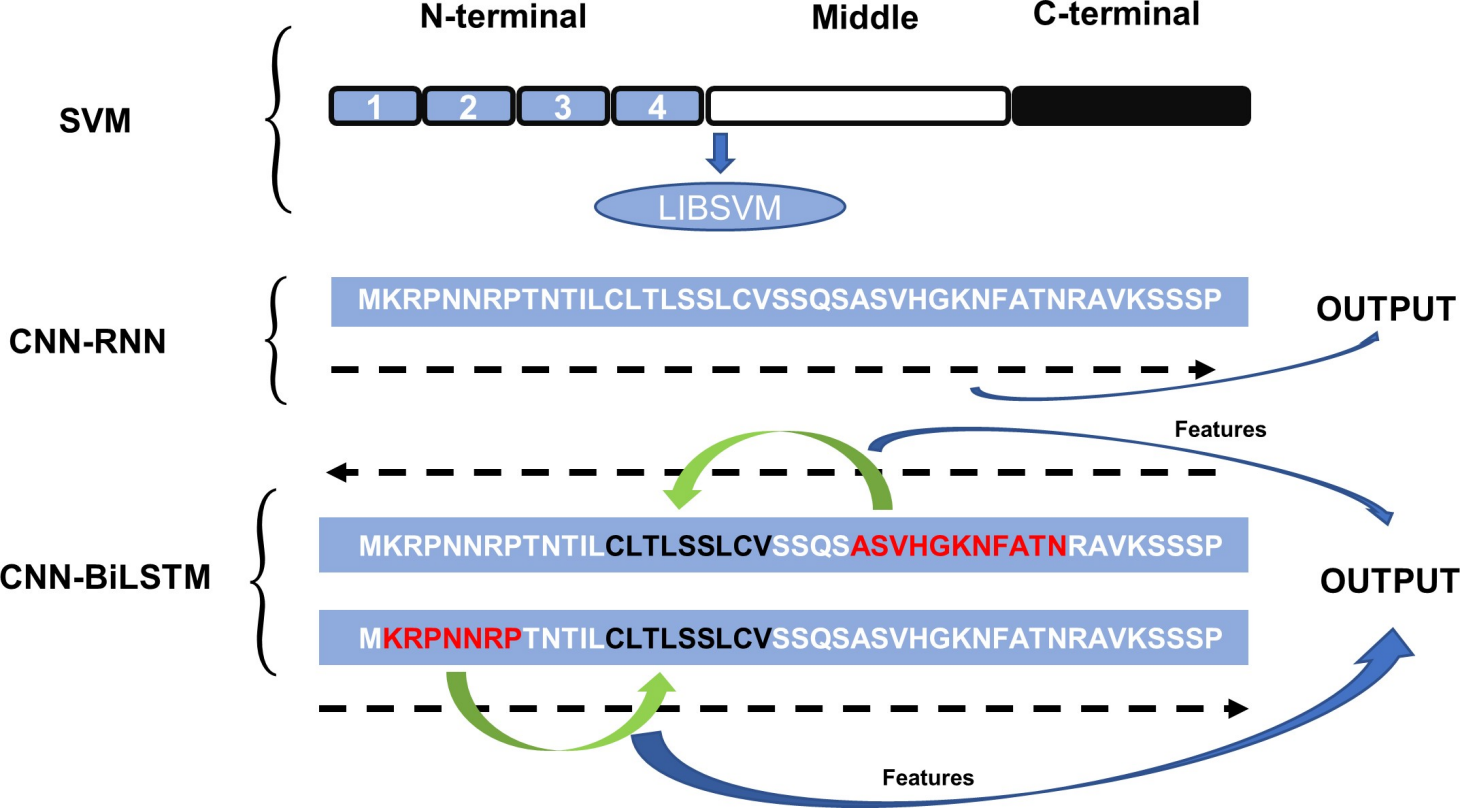
Methods of processing the sequences of different models.

To reveal the differences between the different models in amino acid sequence processing, we compared the CNN-BiLSTM model with the SVM and CNN-RNN models. According to its length, a sequence is divided into the three parts, N, Middle and C, in the method (SVM) proposed in: [15].Then, the sequence features of the three parts are abstracted, and the DNA-binding protein prediction is achieved by the SVM package. In [7], an LSTM was used to process the sequences. During this process, the sequences are scanned unidirectionally. In contrast, the process of scanning sequences in the CNN-BiLSTM model is bidirectional, and the output is constructed by synthesizing the contextual features (see Fig 9 for details).

After the comparison, we find that the CNN-BiLSTM collects more sequence information than do the SVM and CNN-RNN.

### Comparison of score predicted by the different models

**Fig 10:**
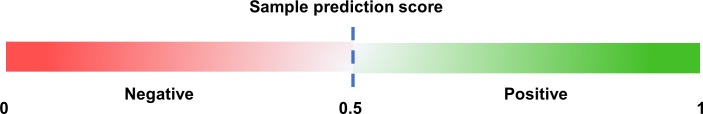
Evaluation criteria for predicted scores.

A deep learning model yields a prediction score for each test sample; when the prediction score is between 0 and 0.5, we consider it as a negative sample. In contrast, when the predicted sample score is between 0.5 and 1, we consider it as a positive sample. The score actually represents the probability that a sample is a positive sample, that is, the closer the score is to 1, the greater the probability that it is a positive sample. CNN-BiLSTM relies on the predictive score to determine whether a sequence is a positive sample (see Fig 10 for details).

**Fig 11:**
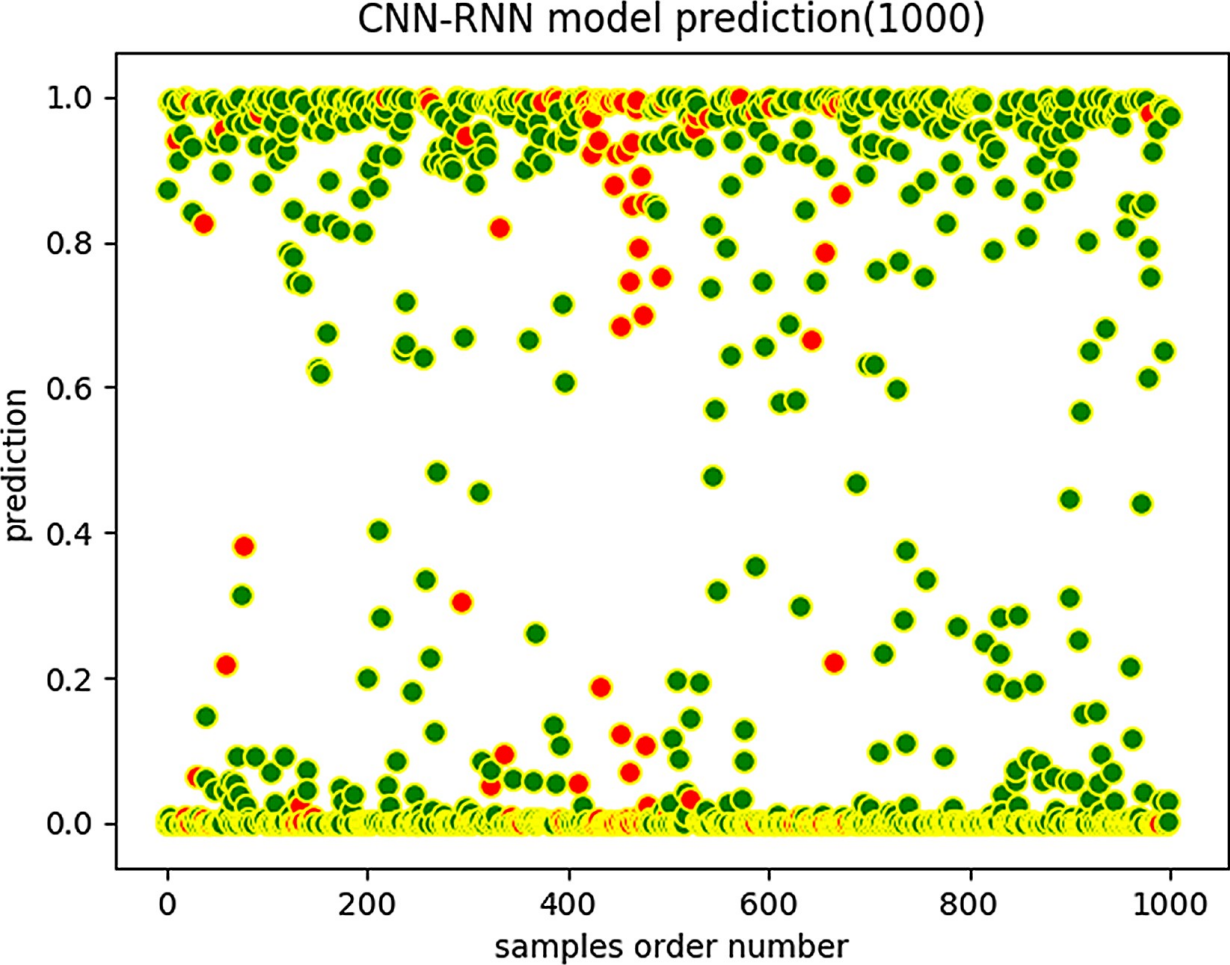
CNN-RNN prediction (1,000).

**Fig 12:**
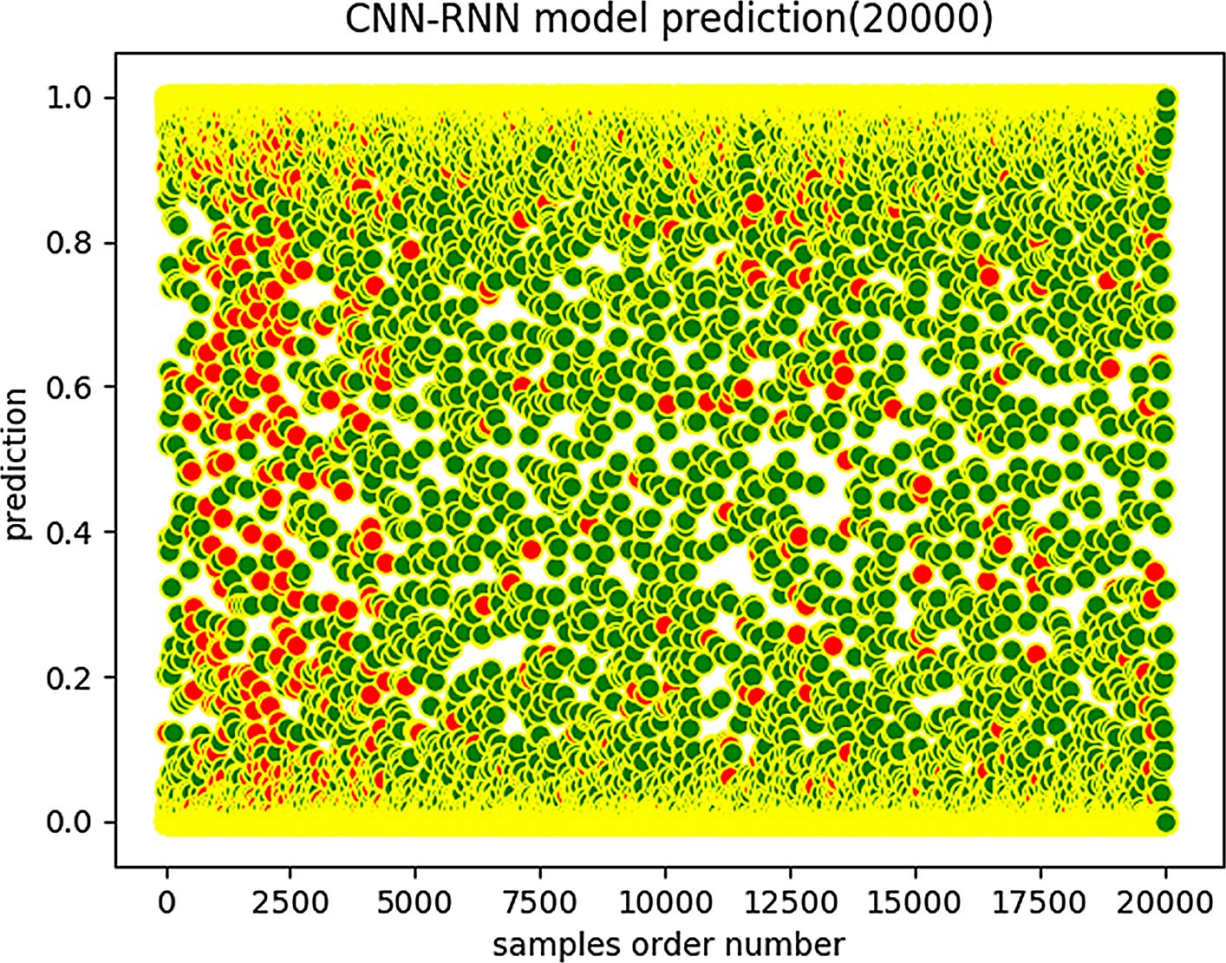
CNN-RNN prediction (20,000).

We trained the CNN-RNN model on the 32,000 samples provided in the literature and obtained a final CNN-RNN model, which does not consider the contextual features of the amino acid sequence. Then, we applied the final CNN-RNN model to the 500-unit test set and the 10,000-unit test set, respectively. It is worth mentioning that these test samples are labeled. We drew the plots of the scores shown in Fig 11 and Fig 12.

In the above diagrams, the horizontal axis expresses the ordinal number of the sequences, and the ordinate expresses the predicted scores of the sequences. A green flag represents a correct sample judgment, and a red sign represents an incorrect prediction.

**Fig 13:**
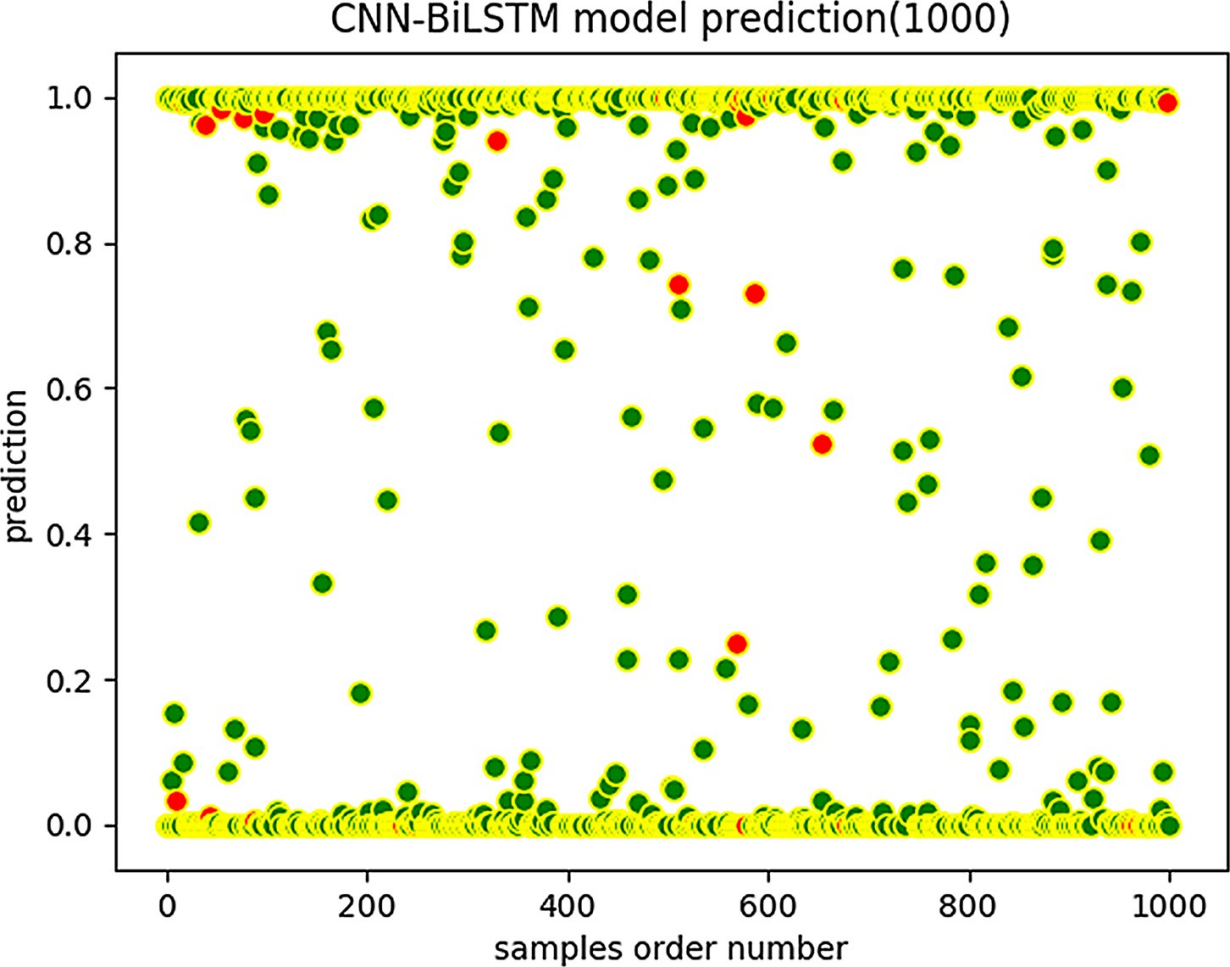
CNN-BiLSTM prediction (1,000).

**Fig 14:**
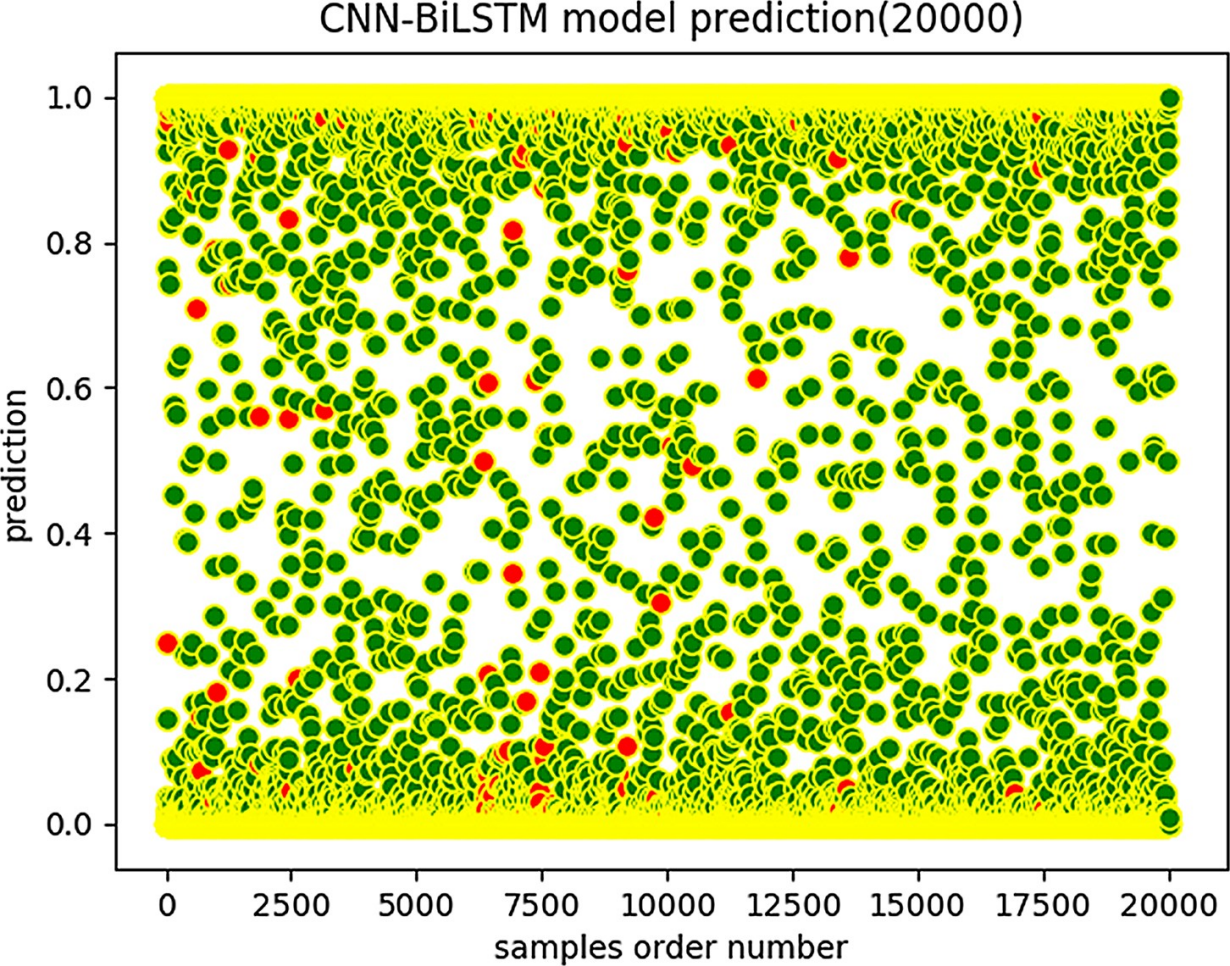
CNN-BiLSTM prediction (20,000).

We then used the same data to train a CNN-BiLSTM model. As with the CNN-RNN results, we also obtained the distribution of the predicted scores of CNN-BiLSTM on the test set as shown in Fig 13 and Fig 14.

We can see that the scores obtained by CNN-BiLSTM are more concentrated in the vicinities of 0 and 1 than are those of the CNN-RNN model, which indicates that the CNN-BiLSTM model has a better predictive score tendency. The prediction score is a probability value. The closer it is to 0 or 1, the more reliable the prediction results are. However, when the prediction score is close to 0.5, the prediction reliability is low. Note that the CNN-BiLSTM model graphs have fewer red marks in the predicted score distribution map than do the CNN-RNN model graphs. Therefore, the CNN-BiLSTM is more trustworthy and robust regarding data fitting and prediction accuracy.

### Model training process visualization

**Fig 15:**
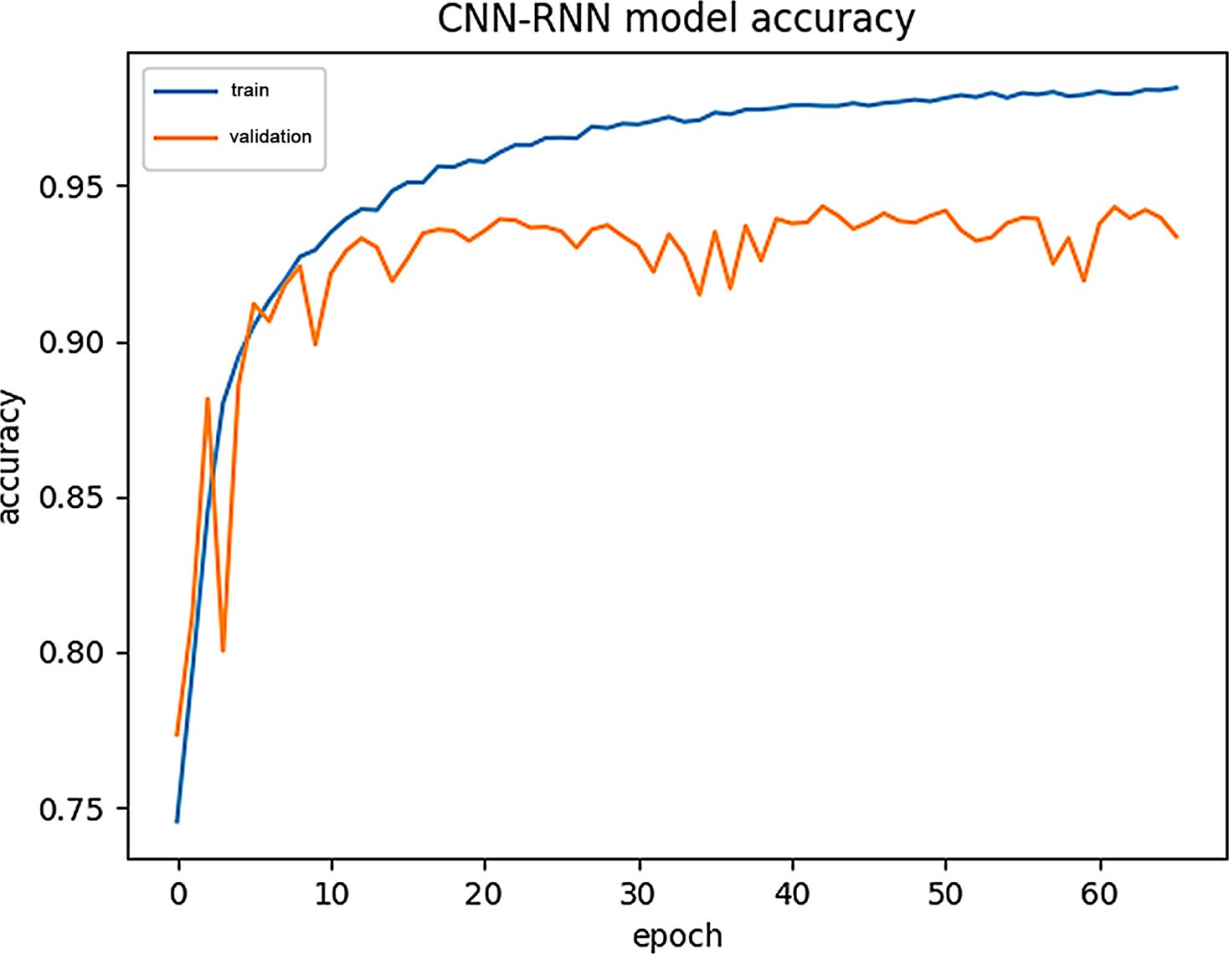
Accuracy variations during CNN-RNN training.

After reconstructing the experiments in [7], we obtained the data for the running process of the model (CNN-RNN). We show the variations of the training accuracy (Train-Acc) and Validation-Acc during the model training process in a single chart. Importantly, Train-Acc refers to the prediction precision of the method on the training set, while Validation-Acc refers to the prediction precision of the method on the validation set used to calibrate the parameter weights of the model. (see Fig 15 for details).

**Fig 16:**
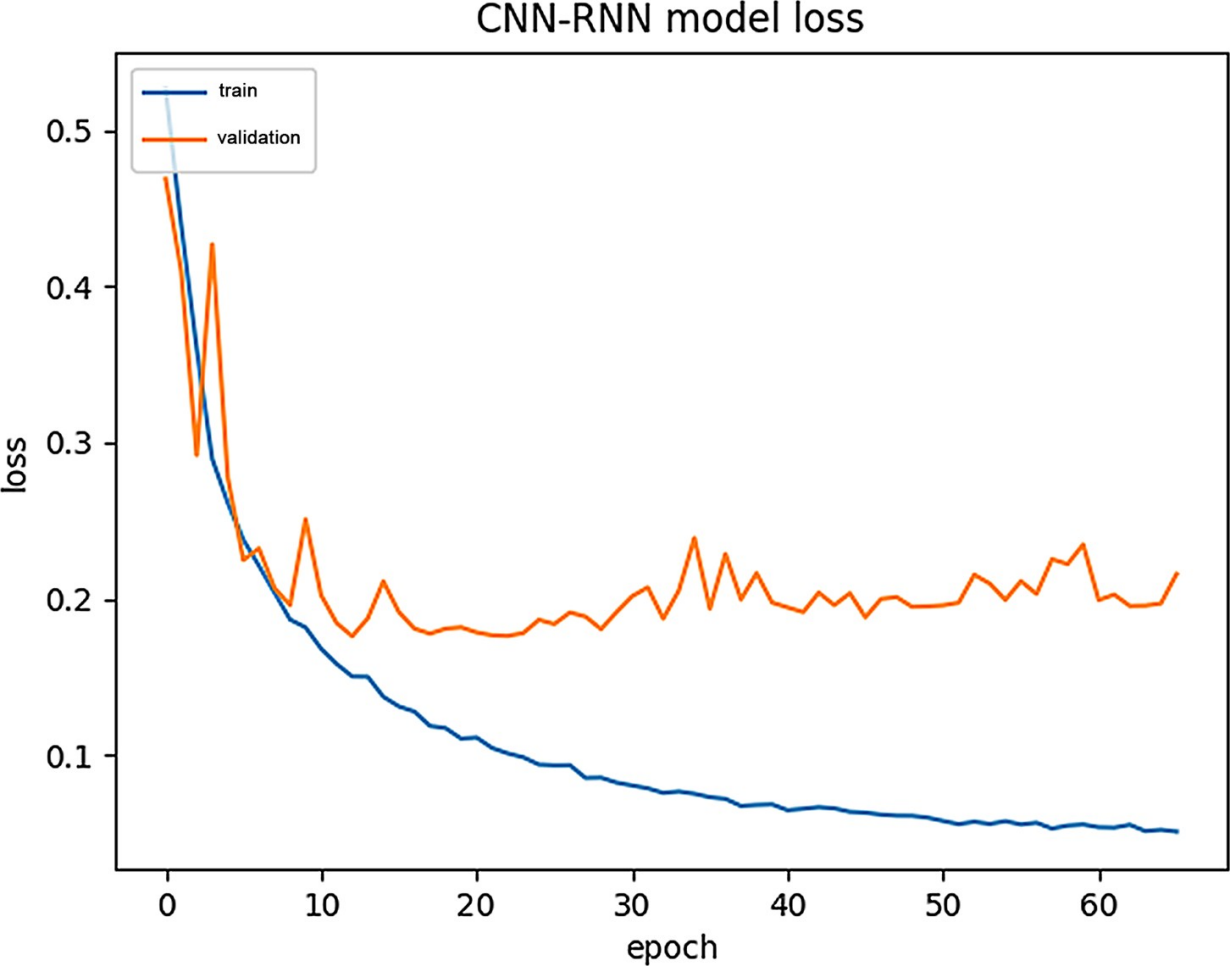
Loss variation during CNN-RNN training.

We also show the variation in the training loss (Train-Loss) and validation loss (Validation-Loss) during the establishment of the above model. (see Fig 16 for details).

**Fig 17:**
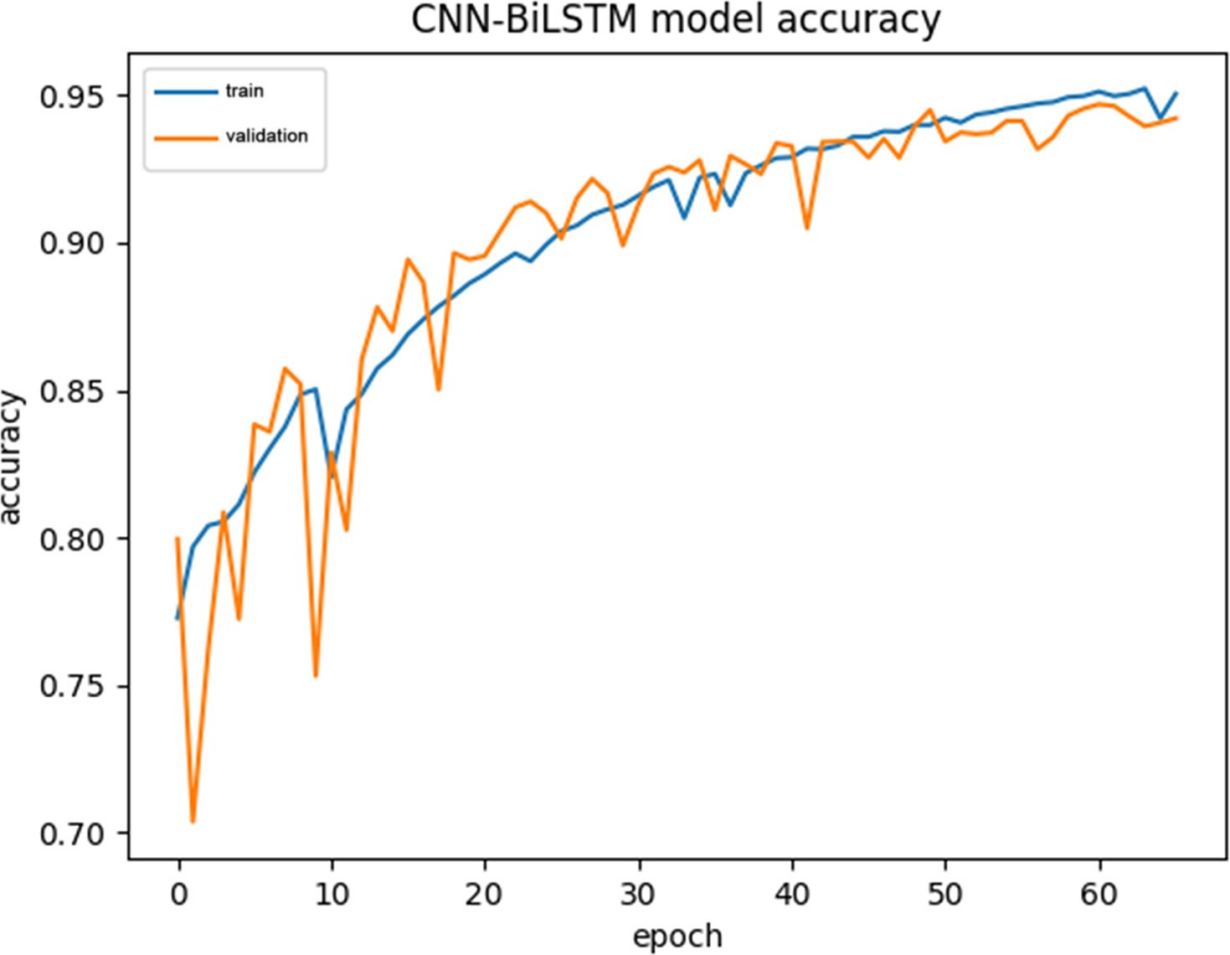
The variation of accuracy in CNN-BiLSTM training.

**Fig 18:**
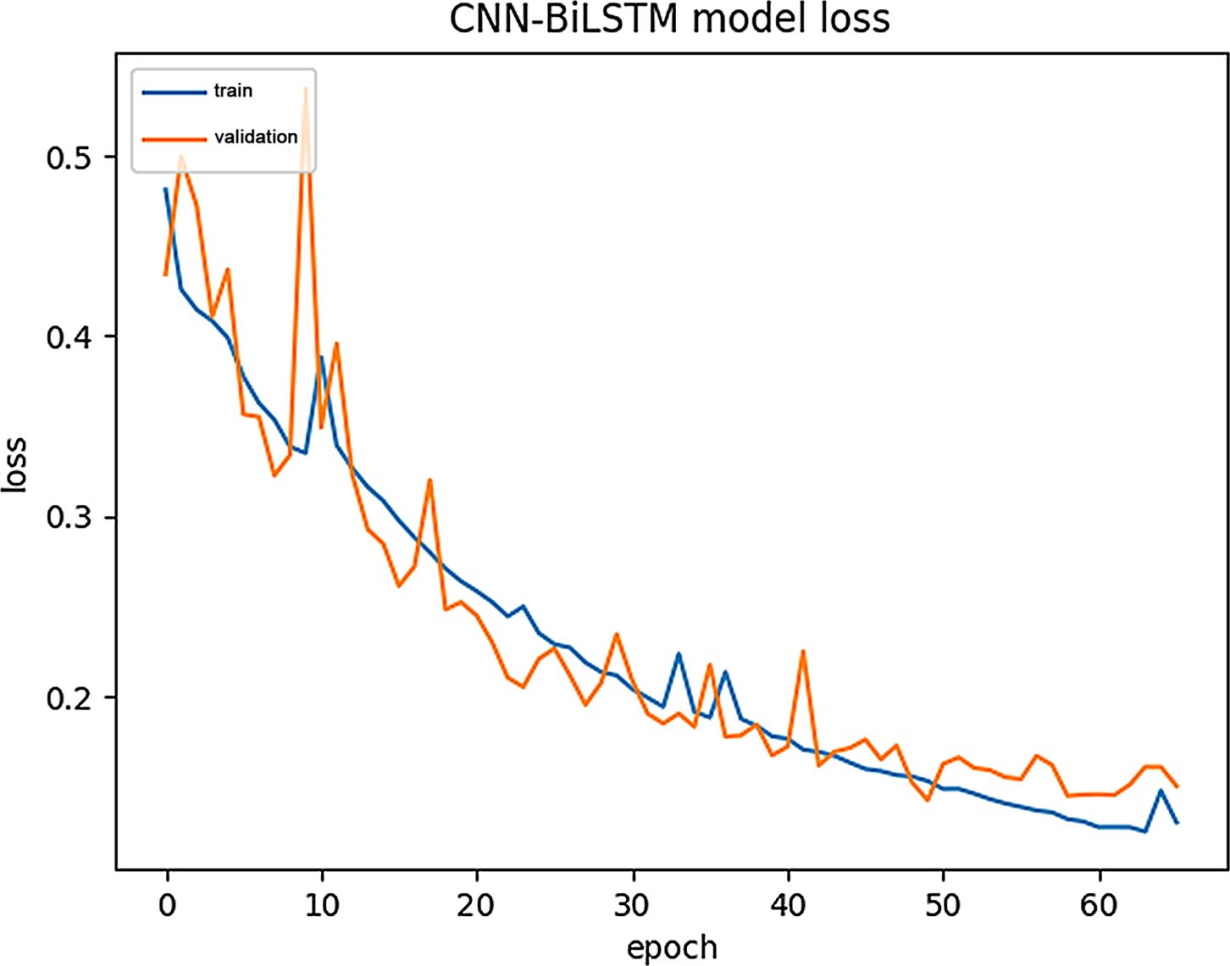
The variation of loss in CNN-BiLSTM training.

For comparison, we used the same data to visualize the CNN-BiLSTM training process. (see Fig 17 and Fig 18 for details).

From Figs 15–18, we can see that the training curve and the validation curve of the CNN-BiLSTM are closer than those of the CNN-RNN, both for Acc and loss. This indicates that CNN-BiLSTM experiences very little overfitting, but the opposite is true in CNN-RNN. Visibly, the training process of CNN-BiLSTM better reflects the real performance of the data.

Additionally, CNN-RNN converges extremely quickly at the beginning of training, but it reaches its upper limit quickly, and the Validation -Loss value (0.2) is still relatively high at this point. In contrast, CNN-BiLSTM converges more slowly, showing a slow climbing trend initially and ultimately reaching a Validation -Loss value close to 0.1. Because CNN-BiLSTM uses a more complex neural network than the other models, it cannot improve the Train-Acc quickly during the initial stage of training, but it does continuously reduce the los value during continuous operation. Although it requires a longer training period, CNN-BiLSTM can capture amino acid sequence features in more detail than can the CNN-RNN.

## Conclusions

The prediction of DNA-binding proteins has been a focus of some computational biologists, and many methods have been proposed successfully. In this paper, we proposed a new deep learning model to distinguish DNA-binding proteins. We combined a CNN and a Bi-LSTM to explore the potential relationships between amino acids that could be used to detect the functional domain of the protein sequence.

Compared with three previous models (SVM, DNABP and CNN-RNN), CNN-BiLSTM achieves a more advanced performance regarding both prediction accuracy and data fitting. In the test for independent samples, the tendency of the predicted scores obtained by CNN-BiLSTM is better than that of CNN-RNN. The use of deep learning methods to discriminate DNA-binding proteins will become more popular. In addition, the method proposed in this paper may have many potential applications elsewhere, such as in predicting plant hemoglobin.
